# Modeling age-specific incidence of colon cancer via niche competition

**DOI:** 10.1371/journal.pcbi.1010403

**Published:** 2022-08-19

**Authors:** Steffen Lange, Richard Mogwitz, Denis Hünniger, Anja Voß-Böhme

**Affiliations:** 1 DataMedAssist, HTW Dresden - University of Applied Sciences, Dresden, Germany; 2 Faculty of Informatics/Mathematics, HTW Dresden - University of Applied Sciences, Dresden, Germany; Oxford, UNITED KINGDOM

## Abstract

Cancer development is a multistep process often starting with a single cell in which a number of epigenetic and genetic alterations have accumulated thus transforming it into a tumor cell. The progeny of such a single benign tumor cell expands in the tissue and can at some point progress to malignant tumor cells until a detectable tumor is formed. The dynamics from the early phase of a single cell to a detectable tumor with billions of tumor cells are complex and still not fully resolved, not even for the well-known prototype of multistage carcinogenesis, the adenoma-adenocarcinoma sequence of colorectal cancer. Mathematical models of such carcinogenesis are frequently tested and calibrated based on reported age-specific incidence rates of cancer, but they usually require calibration of four or more parameters due to the wide range of processes these models aim to reflect. We present a cell-based model, which focuses on the competition between wild-type and tumor cells in colonic crypts, with which we are able reproduce epidemiological incidence rates of colon cancer. Additionally, the fraction of cancerous tumors with precancerous lesions predicted by the model agree with clinical estimates. The correspondence between model and reported data suggests that the fate of tumor development is majorly determined by the early phase of tumor growth and progression long before a tumor becomes detectable. Due to the focus on the early phase of tumor development, the model has only a single fit parameter, the time scale set by an effective replacement rate of stem cells in the crypt. We find this effective rate to be considerable smaller than the actual replacement rate, which implies that the time scale is limited by the processes succeeding clonal conversion of crypts.

## Author summary

Cancer development is a multistep process often starting with a single cell turning into a tumor cell whose progeny growths via clonal expansion into a macroscopic tumor with billions of cells. While experimental insight exists on the cellular scale and cancer registries provide statistics on detectable tumors, the complex dynamics leading from the microscopic cellular scale to a macroscopic tumor is still not fully resolved. Models of cancer biology are commonly used to explain incidence rates but usually require the fit of several biological parameters due to the complexity of the incorporated processes. We employ a cell-based model based on the competition in colonic crypts, to reproduce epidemiological age-specific incidence rates of colon cancer. Due to the focus on the early stage of tumor development, only the time scale in the model has to be calibrated. The agreement between theoretical prediction and epidemiological observation suggests that the fate of tumor development is dominated by the early phase of tumor development long before a tumor becomes detectable.

## Introduction

Cancer development is a multistep process [1, 2] often originating from a single mutated cell [3]. Potential tumor progenitor cells in the tissue accumulate sequentially epigenetic and genetic alterations, which transform them into tumor cells [4]. Initially, these tumor cells can be benign [5, 6], meaning that they do not possess a proliferative fitness advantage, and consequently compete with the original wild-type cells within normal tissue homeostasis [7]. When the first tumor cell acquires a sufficient number of alterations and progresses to a malignant type, i.e., gains a considerable proliferative advantage to the original wild-type cells, a cancer develops [8]: The progeny of the malignant tumor cell spreads via clonal expansion until a sufficiently large cell population is reached to be clinically detectable [9]. A well-known prototype of such a multistage carcinogenesis is the adenoma-adenocarcinoma sequence of colorectal cancer [10, 11], whose intra-tumor heterogeneity suggests that this cancer particularly arises as a single expansion event [12].

Besides this general framework of carcinogenesis, the exact processes by which a tumor develops in the early phase are not known as a tumor is usually only detected after it consists of billions of cells. Mathematical models have been extensively used to elucidate fundamental mechanisms of cancer development and progression on the basis of biological data. One frequently employed interface to link these dynamical models to real-world observations are age-specific cancer incidences from cancer registries. Starting with the multistage Armitage-Doll model [13–15], suggesting that cancer generation is governed by a sequence of rate-limiting events, and multistage clonal expansion model (MSCE) [15–21], which are based on the initiation-promotion-malignant conversion paradigm in carcinogenesis, multitype branching process models [22–24], frailty models [25] as well as other stochastic or regression models [26–30] have been applied to age-specific incidences of various types of cancer. This includes colon [2, 17, 22–24, 29] and colorectal cancer [16, 18, 22, 30], pancreatic cancer [16, 18, 21, 26], gastric cancer [18, 30], esophageal adenocarcinomas (EAC) [18], oral squamous cell carcinomas (OSCCs) [20], prostate cancer [29], gonadal germ cell cancer (most common form of testicular cancer) [28], lung cancer [27], kidney cancer [26], thyroid cancer [19] as well as Hodgkin lymphoma (HL) [25]. Parameters of these models are determined by fitting the models hazard function to the age-specific incidences. A match of the fit and the data is considered to support the validity of the corresponding model. Furthermore, important parameter combinations, which quantify kinetics of malignant progression or clonal expansion before clinical detection, such as growth rates of adenoma, malignant transformation rates, extinction probabilities, as well as sojourn or dwell times, can be estimated via maximum likelihood methods from the fits. While the listed models fit the epidemiological data exquisitely well, they have at least two [30] but usually four [17, 18, 20, 27–30] or more [2, 16, 24, 25] parameters. Thus, there may be issues of identifiability of the parameters from the data for many of these models [21], although the issue is well understood mathematically. The large amount of fit parameters results from the wide range of mechanisms these generic models aim to reflect, often incorporating both the early phase of tumor initiation and the early-to-late phase of clonal expansion. In contrast, the spatial structure of the tissue from which the tumor originates is often neglected, although tissue architecture is known to be crucial for tumor evolution [31]. A recent counter example is a model of colorectal cancer initiation [32], which did not infer the age-specific incidences rates, but recovered the lifetime risk of colorectal adenomacarcinoma, while setting all parameters by experimentally measured rates.

**Fig 1:**
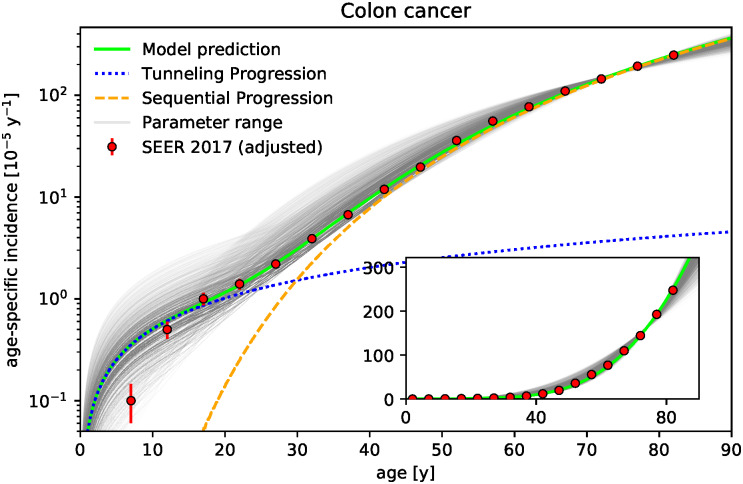
Niche competition in colonic crypts models age-specific incidence rates of colon cancer. Age-specific incidence rates of colon cancer predicted by the model (green and gray lines) agree with epidemiological data (red dots, SEER database [33] adjusted for effects of colorectal screening at >55 years, see Sec C in S1 Text, error bars correspond to 95% confidence interval assuming Poisson distribution and are often smaller than symbol size) over several orders of magnitude (see inset for linear y-scale). For the model prediction the range of the parameters niche size *N*, number of crypts *K*, mutation probabilities *u*, *v*, and probability *γ* of adenoma progressing to adenocarcinoma are taken from the literature, see Table 1, while the time scale set by the effective replacement rate λ is calibrated based on the epidemiological data. Sensitivity on parameters is illustrated by variation of the parameters within the reported ranges (gray lines, opacity scaled inversely with corresponding goodness-of-fit ∼1/*χ*^2^ for clarity). Exemplary parameter set with *N* = 8, *K* = 2 ⋅ 10^7^, *u* = 7.13 ⋅ 10^−6^, *v* = 1.75 ⋅ 10^−6^, and *γ* = 9.4% is highlighted in green. Decomposition of incidence rates predicted by the model into incidences with and without precancerous lesions (sequential and tunneling progression, orange dashed and dotted blue line respectively for the exemplary parameter set) confirms different origins of cancer at young and older ages. The effective replacement rate λ is found to be considerably smaller than the actual replacement rates (λ = 0.016 y^−1^ per stem cell for the green curve and λ = 0.024±0.01 y^−1^ for the gray curves).

**Fig 2:**
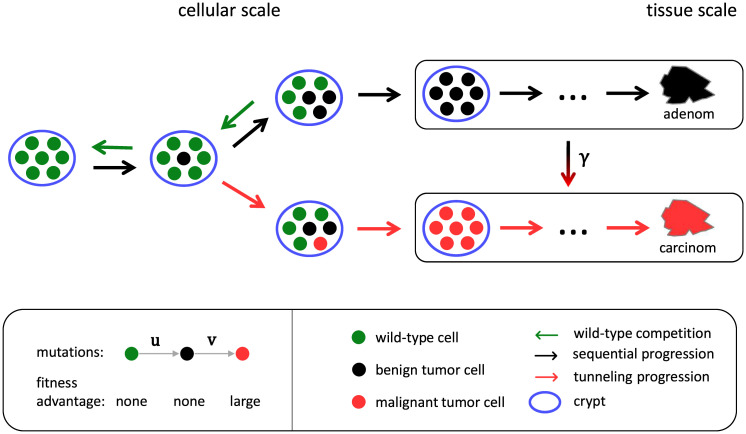
Illustration of the dynamics of the niche-competition-based pretumor progression model. Wild-type cells within the niche of a colonic crypt can progress to benign tumor cells, which can further progress to malignant tumor cells, during proliferation with mutation probabilities *u*, *v*. While wild-type and benign tumor cells neutrally compete by replacing each other in the niche with rate λ, malignant tumor cells rapidly dominate the niche. We assume that once no more wild-type cells are present in the niche (fixation or clonal inversion) tumor cells establish within the tissue via clonal expansion. Consequently, there are two possible progression pathways: (i) The niche consists of malignant tumor cells at fixation and an adenocarcinoma grows from the niche (tunneling progression). (ii) The niche consists of benign tumor cells at fixation and the resulting tumor progresses with probability *γ* at some point during growth to an adenocarcinoma (sequential progression, precancerous lesion).

**Fig 3:**
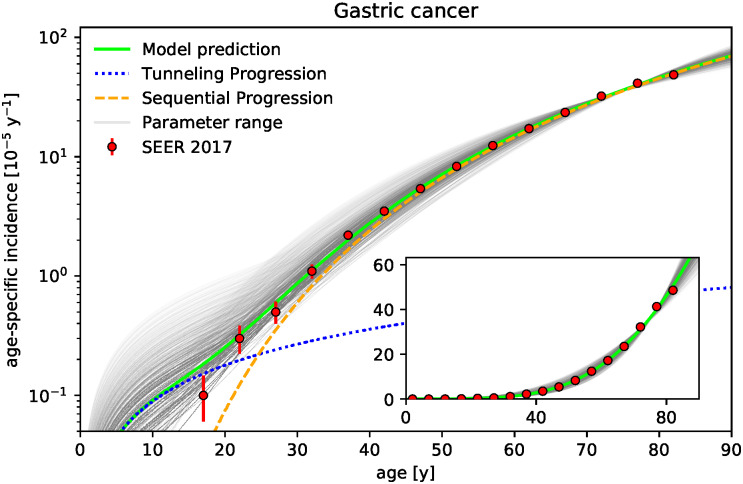
Niche competition could model age-specific incidence rates of gastric cancer. Age-specific incidence rates of gastric cancer predicted by the model show correspondence to epidemiological data (SEER database [33], displayed curves and points analogous to Fig 1). Since parameters are not available as detailed as for the colon, the range of the parameters *N*, *u*, *v*, and *γ* are assumed to match the ranges for the colon, see Table 1, while the range of the number of gastric glands *K* = 4–16 ⋅ 10^6^ is estimated as described in the text. Exemplary parameter set with *N* = 7, *K* = 8 ⋅ 10^6^, *u* = 4.4 ⋅ 10^−6^, *v* = 1.75 ⋅ 10^−6^, and *γ* = 5% is highlighted in green. The effective replacement rate λ is found to be considerably smaller than replacement rates of colonic crypts (λ = 0.014 y^−1^ per stem cell for the green curve and λ = 0.02±0.01 y^−1^ for the gray curves illustrating the parameter range).

**Fig 4:**
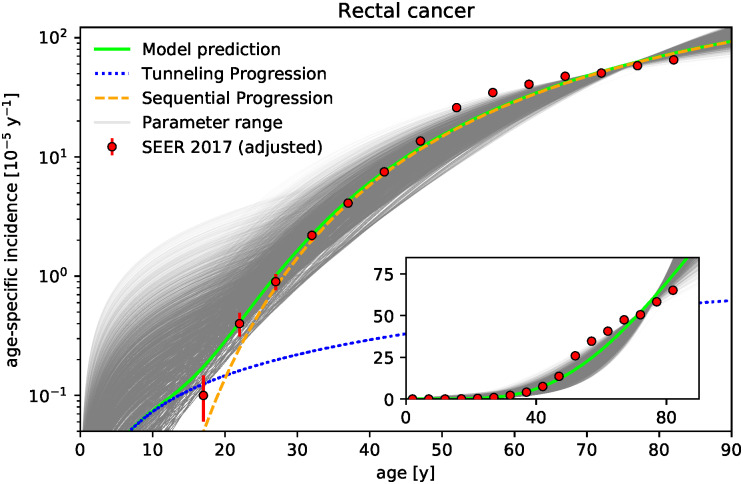
Niche competition model corresponds well to age-specific incidence rates of rectal cancer for age groups below 50. Age-specific incidence rates of rectal cancer predicted by the model shows rough correspondence with epidemiological data (SEER database [33], displayed curves and points analogous to Fig 1). Same parameters as for the the colon are assumed, see Table 1, except for the number of crypts *K* which is assumed to be ten times smaller *K* = 10^6^ − 2 ⋅ 10^6^ reflecting that the rectum has on average a tenth of the length of the colon. Exemplary parameter set with *N* = 8, *K* = 10^6^, *u* = 1.75 ⋅ 10^−6^, *v* = 1.75 ⋅ 10^−6^, and *γ* = 5% is highlighted in green. The effective replacement rate λ is found to be considerably smaller than replacement rates of colonic crypts (λ = 0.05 y^−1^ per stem cell for the green curve and λ = 0.04±0.02 y^−1^ for the gray curves illustrating the parameter range).

Our goal is to demonstrate that the competition between wild-type and tumor cells in niches in the early phase of tumor initiation may be a major mechanism for the fate of tumor development. For this, we develop a cell-based stochastic model which represents the pretumor competition between wild-type and tumor stem cells in colonic crypts and thus both focuses on the early-phase of tumor initiation as well as explicitly incorporates the spatial structure of the tissue. The model reproduces the age-specific incidences of colon cancer from the Surveillance Epidemiology and End Results (SEER) database quantitatively, see Fig 1. The model predictions agree with the epidemiological data not only in the regime of older ages but also down to young ages, where incidences are several orders of magnitude smaller. This correspondence supports the recently proposed notion that the fate of tumor development may be determined in the early phase of tumor development long before a tumor becomes detectable [34]. Since the model explicitly distinguishes between (i) adenocarcinoma and (ii) adenoma, which progressed to adenocarcinoma, see sketch of the model in Fig 2, we additionally predict the fraction of incidences corresponding to either type. These fractions are in agreement with the common clinical estimates that more than 95% of colon adenocarcinomas arise from colonic polyps and that the fraction of benign tumor is over 99% [35]. In particular, we quantify how the fraction of incidences resulting from progressed adenoma increases with age, which supports the relevance of colorectal cancer screening at older age. We emphasize that all parameters of the model correspond directly to known physiological parameters and are consequently set by previously reported values. Only the effective stem cell replacement rate within a crypt is used as a single fit parameter to set the time scale. We find this effective replacement rate to be considerably smaller than the actual replacement rate, which implies that the time scale is limited by the processes succeeding clonal conversion of a crypt. The model is additionally applied to gastric and rectal cancer, see Figs 3 and 4, whose tumor-originating cells are proposed to be also compartmentalized into niches.

## Materials and methods

### Cancer screening data

The Surveillance Epidemiology and End Results (SEER) research database comprises cancer incidences and at-risk population data in the US [33]. We consider age-specific incidence rates of colon cancer as reported in the most recent SEER dataset 2013–2017. For the age-specific incidence rates in the SEER report the number of diagnosed cancers, as a function of age, is compiled and then divided by the corresponding total population at risk. The incidence rates are reported in eighteen 5-year age groups and we assign rates to the midpoint of these groups. The last age group 85+ is excluded due to rapidly declining person-years after age 85. We estimate the confidence interval of the rates by assuming a Poisson distribution of the incidences and compute the range containing 95% of the incidences based on the US Standard population 2000 [36] in each age group.

However, the main source of uncertainty is not the statistical error but the secular trends of incidence rates observed for colorectal cancer: While the overall incidence of colorectal cancer has been decreasing since 1998 due to a decrease in the age groups >50 years, the incidence for men and women younger than 50 has been rising, in particular for adolescents and young adults [37, 38]. The incidence rates exhibit these age-group-dependent trends both over calendar year and birth cohort, see also Fig B in S1 Text. The decrease is attributed to the widespread uptake of colonoscopy screening (and consequent resections) for people above 50 [37, 39–41]. To account for the effect of colorectal cancer screening, we consider several reasonable adjustments of the incidence rates from the SEER 2017 dataset. As representative case, we display the results for colon and rectum in Figs 1 and 4, respectively, for which the rates of all age groups above 55 years have been increased by a factor 4/3. This adjustment is based on estimates that the annual incidence of colorectal cancer at ages >50 between 2000 and 2015 is reduced on average by ∼25% due to screening [40, 42]. Results for other adjustments (including the case of the unaltered rates) are reported in Sec C in S1 Text. For the colon, we find for each adjustment a similarly good correspondence between the epidemiological incidence rates and our model prediction. Mostly, the fitted effective replacement rate for a particular parameter set is modulated by the choice of the adjustment. Furthermore, we find agreement of our model predictions with incidence rates of gastric cancer, whose secular trends are far less pronounced.

At ages younger than 50 colorectal screening occurs rarely. While the upward trend of the incidences at these younger ages is less understood, two opposing effects have been implicated: On the one hand, the apparent rise in incidences below 50 years may be attributed to life-style changes [37, 43] an effect not incorporated in our biological model. On the other hand, recent results support the presence of a large undetected preclinical case burden <50 years, which is not reflected in the rates of colorectal cancer observed in the SEER registries [44]. The latter is consistent with the fact, that people younger than 55 are more likely to be diagnosed with late-stage disease, largely due to sometimes for years delayed follow-up of symptoms [43]. This implies that cancer incidences are either missed at younger ages or wrongfully assigned to older age groups, whose rates they barely impact as the age-specific rates increase significantly with age. Since the net impact of these two opposing effects is unknown, we do not adjust incidence rates below 55 years, similar to Ref. [18]. Note that external factors, like life-style changes and screening effects, can be indirectly captured by adjustments of the observed incidence rates for age, birth cohort, and time period effects, for instance via age-period-cohort (APC) epidemiological models [15, 17, 18, 20, 45]. However, while such statistical adjustments compensate for secular trends, they do not allow to determine what the incidence rates would be in the absence of these external factors. Consequently, such adjustments are often not invoked when comparing incidence rates to biological models of cancer development, in particular for colon cancer [2, 22–24, 29, 32].

Note that incidences of colorectal cancer differ between sexes [2, 16–18, 30, 45], races [45] as well as left-(distal) and right(proximal)-sided cancers [17, 40]. Since we focus with our model on the net effect of basic biological processes and thus do not incorporate mechanisms accounting for these differences, e.g., screening differences which account for almost half of the disparity between colorectal incidences in white and black populations [37], we consider the total number of incidences regardless of sex, race or site, as previous model approaches [22, 24, 32].

### Pretumor progression model based on niche competition

In order to test the hypothesis, that the fate of tumor development is determined by niche competition in the early phase of tumor development, we predict the incidence rates of colon cancer by a mathematical model based on the tumor initiation of the adenoma-adenocarcinoma sequence. The tumor-originating cells of colon cancer are known to be multipotential stem cells located in a niche at the base of intestinal crypts, also called intestinal glands, which are finger-like invaginations on the surface of the intestine [11, 12, 46]. On average such a stem cell divides asymmetrically, resulting in one daughter cell which remains as stem cell in the niche and another daughter cell, which is committed to differentiation and leaves the crypt, such that the number of stem cells in the niche is always roughly conserved. However, the progeny of one stem cell can effectively replace the progeny of another whenever symmetric divisions occur, that is one stem cell divides into two stem cells while another divides into two differentiated cells, which leave the crypt. During division stem cells can mutate and thus gradually accumulate alterations which turn them into tumor cells. Tumor cells compete with the original stem cells via replacement in the niche and thus may at some point dominate the entire niche (niche succession or clonal conversion). The so fixated tumor cells may then grow across the intestinal epithelium by crypt fission and clonal expansion of tumor cells from the converted crypt and consequently a tumor develops. If tumor cells are benign at that point, a premalignant lesion (adenoma) develops, which may advance to cancer (adenocarcinoma) given a tumor cell progresses to a malignant type (sequential progression). If tumor cells are already malignant during fixation in the niche, an adenocarcinoma grows directly (tunneling progression).

We emulate this multistep process using a cell-based Moran model representing the competition between wild-type stem cells and tumor cells in the niche [34, 47], see Fig 2 for illustration and Sec A in S1 Text for details: We consider a fixed number *N* of cells in the niche, all of which are initially wild-type stem cells. During proliferation a wild-type cell can mutate with probability *u* to a benign tumor cell, which in turn can mutate with probability *v* to a malignant tumor cell. Wild-type cells and benign tumor cells compete by replacing each other in the niche with rate λ. Eventually, via this competition, existing benign tumor cells either go extinct or they replace every wild-type cell in the niche. In the latter case, the tumor cells are considered to spread and develop a clinically detectable adenoma. From the Moran model corresponding to these dynamics, we obtain the adenoma probability *P*_*a*_(*t*) that an adenoma has developed from the crypt until age *t*, see Eq. (S5). Alternatively, during competition in the niche, any benign tumor cell can acquire malignancy. Due to the proliferative fitness advantage thus acquired, a malignant tumor cell rapidly dominates the niche and consequently an adenocarcinoma develops directly. From the Moran model, we obtain the carcinoma probability *P*_*c*_(*t*) that an adenocarcinoma has developed from the crypt until age *t*, see Eq. (S6).

Thus, there are two possible origins of colon cancer in the model: (i) Firstly, tunneling progression, which means an adenocarcinoma develops directly from a single niche with probability *P*_*c*_(*t*). (ii) Secondly, an adenoma develops in a single niche with probability *P*_*a*_(*t*) and progresses with probability *γ* to an adenocarcinoma during clonal expansion. Therefore, the probability that cancer has developed from the crypt until age *t* via this sequential progression is *γP*_*a*_(*t*). Consequently, the combined probability that an adenocarcinoma develops from a single crypt until age *t*, either with benign precursor stage or directly, is *P*_*c*_(*t*) + *γP*_*a*_(*t*). Inversely, the probability that a crypt has not given rise to a cancerous tumor until age *t* is 1 − *P*_*c*_(*t*) − *γP*_*a*_(*t*). Taking into account that there exist *K* crypts in the colon and a single one suffices as origin of a tumor, the probability *S*(*t*) that a human has not contracted colon cancer until age *t* is

$$\begin{array}{c} S\left(t\right)={\left(1-P_{c}\left(t\right)-\gamma P_{a}\left(t\right)\right)}^{K}\;,\;S\left(0\right)=1\;. \end{array}$$ (1)

From this survival function *S*(*t*), the model’s age-specific incidence rate *R*(*t*), or hazard function, is computed

$$\begin{array}{c} \begin{array}{cc} \frac{d}{dt}S\left(t\right) & =-R(t)S(t)\\ R(t) & =-\frac{d}{dt}\text{ln}\;S\left(t\right) \end{array} \end{array}$$ (2)

The six parameters *N*, *K*, *u*, *v*, λ and *γ* of the model are all directly measurable, independently of the emerging age-specific incidence rates, and their values have already been determined or estimated in previous experiments, see Table 1. Note that a simpler version of the model has already been used to estimate the competition range for several types of solid tumors from the fraction of observed benign tumors [34] and to evaluate the spontaneous tumor regression in pilocytic astrocytoma [47].

**Table 1:** Table of previously reported ranges of the parameters used in the niche model. The upper and lower boundaries as well as the expected value of each parameter *K*, *v*, *u*, or *γ* are used for the parameter variation, see gray lines in Fig 1, by which the effect of the parameter range on the prediction of the model is estimated.

Parameter			previously reported range
niche size	*N*		5…7…15 [52–54]
number of crypts	*K*	[10^7^]	1…1.5…2 [55, 56]
mutation probabilities	*u*, *v*	[10^−6^]	1.75…4.4…7.13 [52]
fraction of adenomas progressing to carcinomas	*γ*	[%]	1.5…5…9.4 [57, 58]
stem cell replacement rate	λ	[y^−1^stem cell^−1^]	0.3…1…2.0 [52, 54, 59]

The model employs some reasonable simplifications: While colon cancer increases malignancy gradually in seven steps [48], we only regard the last benign alteration and the first malignant alteration. In particular for colon cancer, more than two steps or more than one type of genomic instability are not expected to enhance the agreement between a progression model and the epidemiological data [2]. Furthermore, we do not assume a particular spatial arrangement of the stem cells in the niche, but rather use two limiting cases of either space-free (all-to-all competition) or one-dimensional cell arrangement (competition only with two neighbors). Since the model’s probabilities *P*_*a*_(*t*) and *P*_*c*_(*t*) depend monotonously on the number of cells which can compete with each other [49], the incidence rates for any cell arrangement should lie in between these two limiting cases. Note that typically neighboring replacement is assumed, consistent with observations from genetic lineage tracing in mouse and recent observations of epidermal stem cells [50]. However, for the small number *N* of stem cells in a colonic crypt these two limiting cases yield almost matching results. Furthermore, while the model allows explicitly for two different mutation probabilities *u*, *v*, we use the same parameter range for both, see Table 1, although the benign mutation may allow the malignant mutation to occur more easily *v* > *u*. Finally, the model neglects the growth kinetics after tumor cells fixate in a niche. The details of this growth may be very complex and the length of the adenoma-adenocarcinoma interval depends on size, morphology and pathological type of the adenoma [51] but the kinetics have been estimated by models with clonal expansion [18]. Note that a single converted crypt does not directly lead to a carcinoma but rather additional processes like crypt fission and evolutionary and adaptive processes that establish a tumor micro-environment and a permissive immune-ecology are critical for the spread and fixation of the mutation in the tissue. We take these processes only implicitly into account by using the replacement rate λ in the model as an effective parameter setting the time scale. Thus, this effective rate is expected to be smaller than the actual replacement rate, reflecting the additional time to fixation in the tissue and diagnosis. This smaller effective replacement rate also allows to compensate for the simplified competition after occurrence of a single malignant tumor cell within the niche. This is again reasonable due to the small number *N* of cells a malignant cell has to outcompete in a colonic crypt. Finally, the model, while conceptually very different, produces age-specific incidence rates with features typically known from multistage clonal expansion models (MSCE) [16, 17, 21], that is incidence rates exhibit a power law at young age and transition with increasing age to an exponential increase, followed by a linear increase until asymptotically becoming constant, see Sec A in S1 Text for details. Note that MSCE models include an early phase without expansion [18], resulting in a non-zero probability that cancer arises within a single crypt or from a very small number of initiated cells, which corresponds effectively to a fixation before proliferation of crypts.

## Results

We apply our pretumor progression model to age-specific incidence rates of colon cancer, see Fig 1, adjusted for effects of colorectal screening, see Sec C in S1 Text. We set the model’s parameters as reported for the colon in the literature, see expected values in Table 1, and calibrated the effective replacement rate λ by the epidemiological data, see Sec B in S1 Text. The resulting prediction of the model agrees with the age-specific incidence rate of the most recent SEER data not only in the regime of older ages, see rates on linear scale in inset of Fig 1, but also down to young ages, where incidences are more than two orders of magnitude smaller, see rates on logarithmic scale in the main panel of Fig 1. The only exception is the single incidence rate at 5–9 years, for which the prediction is about four times higher than the data. This discrepancy may result from the mentioned issue of delayed diagnosis, which may have a particular impact at this young age and small incidence (5-times smaller than incidence in next age group 10–14).

The robustness of the model prediction within the parameter ranges taken from the literature is checked by a coarse parameter variation: For all possible parameters sets, for which each of the parameters *K*, *u*, *v*, *γ*, either assumes the lower or upper limit or expected value as reported in the literature, see Table 1, and for which *N* assumes any integer 5–15, the effective replacement rate λ is fitted to the epidemiological data resulting in an ensemble of model predictions. For illustration, this ensemble is displayed by gray lines in Fig 1, where for clarity the opacity of each line scales inversely with the goodness-of-fit. It turns out, that fixing any of the parameters *K*, *v*, *u*, or *γ* to one of its three values while varying all other parameters, leads to an ensemble of model predictions whose average effective replacement rate, life-time fraction of benign tumor and goodness-of-fit is virtually the same in either case. In contrast, effective replacement rate and goodness-of-fit strongly depend on the niche size *N*. In the following, we consider the parameter sets whose goodness-of-fit is less than hundred times bigger than the smallest occurring value, which corresponds roughly to the gray lines visible in Fig 1. Within this ensemble, the niche sizes are predominantly *N* = 6–10 with an average *N* = 8±1.8, meaning that the model fits better for the average or lower limit of the niche size. The fact that the niche size *N* has such an impact on the predictions but not the number of crypts *K*, which also affects the total number of stem cells, highlights the crucial role of the competition in the niche, which is only affected by *N* but not *K*.

Furthermore, we estimate from the model the life-time fraction of adenocarcinoma, which arise from colonic polyps, as *γP*_*a*_(*T*)/(*P*_*c*_(*T*) + *γP*_*a*_(*T*)) = 96.4±1.3% (*T* = 85 y) and the life-time fraction of benign tumor as *P*_*a*_(*T*)/(*P*_*c*_(*T*) + *P*_*a*_(*T*)) = 99.7±0.1% in agreement with the corresponding clinical estimates of >95% and 99% [35].

In the main panel of Fig 1, the incidence rates from the model are additionally decomposed into the contribution from (i) tunneling progression without precancerous lesions −d/d*t* ln(1 − *P*_*c*_(*t*))^*K*^ and (ii) sequential progression −d/d*t* ln(1 − *γP*_*a*_(*t*))^*K*^. The incidences at younger age are dominated by the former type, while they result almost solely from the latter one for ages beyond 40. Furthermore, the incidence rates from the tunneling progression exhibit a linear slope ∼*t* while the sequential progression leads to a much steeper course, which resembles a Weibull distribution. These features of the model’s dynamics are consistent with the fact that early onset gastrointestinal cancers commonly arise without precancerous lesions while adenomatous lesions are more common in older patients and that carcinogenesis accelerates after the age of 40 [30, 60, 61]. Note that the probability *γP*_*a*_(*t*) should be understood as an upper limit for sequential progression from a single crypt as some tumors may turn malignant during tumor growth or go extinct before the adenoma becomes detectable. Consequently, the probability *P*_*c*_(*t*) is a lower limit of the fraction of tunneling progression. This implies that the age at which the contributions of both progressions match *P*_*c*_(*t*) = *γP*_*a*_(*t*) is underestimated by the model in Fig 1.

The ensemble of model predictions covers a range of effective replacement rates 0.01–0.06 y^−1^ per stem cell with an average λ = 0.024±0.01 y^−1^ per stem cell. While an effective rate smaller than the actual rate is in principle expected due to the neglected timescale between fixation in the crypt and clinical detection, this effective rate is considerably (factor 5–200) smaller than the physiological replacement rates reported in the literature, see Table 1 [52, 54]. This suggests that, while the course and composition of the age-specific incidence rates seems to be determined by the competition in the crypt, the time scale is predominantly set by the processes succeeding the conversion of crypts. Note that the physiological replacement rate itself has been recently updated [52, 54] by two orders of magnitude compared to previous estimates, which puts the replacement rate close to the cell division rate [53, 62]. Finally, note that we adjusted the epidemiological incidence rates for effects of colorectal screening via different estimates and observe similar correspondence between model and data in all cases, see Sec C in S1 Text.

The model is additionally applied to gastric cancer, whose tumor-originating cells are proposed to be also compartmentalized into niches, see Fig 3. In the context of gastric cancer, it is known that gastric corpus and antrum have distinct stem cells regarded as the tumor-originating cells, which are also structured in stem cell niches within gastric glands [63]. Since the corresponding parameters are not available as detailed as for the colon, the range of the parameters *N*, *u*, *v*, and *γ* are assumed to match the ranges for the colon, see Table 1, while the number of gastric glands *K* = 4–16 ⋅ 10^6^ is estimated from the approximate surface of the stomach ∼800 cm^2^ and the density of glands 135 mm^−2^ [64]. We find a good agreement between model prediction and epidemiological data, see Fig 3. The obtained effective replacement rate λ = 0.02±0.01 y^−1^stem cell^−1^ is similar to the value obtained from the model for the colon, see Fig 1.

The model is additionally applied to rectal cancer, whose tumor-originating cells are also compartmentalized into niches, see Fig 4. For rectal cancer, it is reasonable to assume the same parameters as for the colon, see Table 1, except for the number of crypts *K*. Since the rectum is on average one order of magnitude shorter than the colon, the number of niches *K* is assumed to be ten times smaller *K* = 10^6^ − 2 ⋅ 10^6^. The resulting prediction of the model displays a good qualitative correspondence for the age-groups below 45, see Fig 4. However, there are considerable deviations in the age groups above 50 years. Still, the model predictions displays a rough visual correspondence to the data, capturing essential, qualitative characteristics, which is not self-evident considering that only a single parameter has been fitted. In addition, the data points at older age groups may be problematic due to an underestimated impact of rectal screening. Risk reduction is known to vary by subsite of the colon and rectum [42] while the estimates of this risk reduction are for colorectal cancer [39, 40, 42], whose incidences are dominated by incidences of colon cancer. Indeed, assuming higher reduction of annual incidences for rectal cancer due to screening leads to substantially better correspondence between model and data, see Figs G and H in S1 Text. In any case, according to our model prediction the incidences of the rectum are dominated by sequential progression even at small ages. The obtained effective replacement rate λ = 0.04±0.02 y^−1^ per stem cell is again considerable smaller than the range reported for the colon. In particular, the obtained effective replacement rates λ for both rectal and gastric cancer are similar to the one obtained for colon cancer. However, compared to colon cancer, contribution of tunneling progression to the incidence rates of rectal and gastric cancer result are much smaller with deviations from the sequential progression only visible below 25 years.

## Discussion

We employed a Moran model representing the pretumor competition between wild-type and tumor stem cells in colonic crypts to quantitatively reproduce age-specific incidences of colon cancer. Additionally, the model predicts the fraction of incidences corresponding to either (i) adenocarcinoma or (ii) benign polyps, which progressed to adenocarcinoma, and we find these fractions in agreement with common clinical estimates. Furthermore, the age-dependency of these fractions is consistent with the occurrence of precancerous lesions in different age regimes. In particular, this highlights the importance of cancer screening after the age of 40. Note that all growth and clonal expansion processes after the fixation of tumor cells in the tumor originating niche are not explicitly incorporated into the model, but only implicitly contained in an effective replacement rate. We find this effective rate to be substantially lower than the actual physiological replacement rate [52, 54]. Thus, the agreement between model and epidemiological data supports the notion that the fate of tumor development may be majorly determined by the early phase of tumor development, while the time scale of tumor development is primarily set by processes of clonal expansion and tumor growth.

We emphasize that our model does not suggest, that a single converted crypt results inevitably into a macroscopic carcinoma, which would be biologically untenable. Rather all processes between conversion of crypts and diagnosis of a macroscopic malignancy are omitted, since the purpose of the model is to estimate the impact of the earliest phase of tumor development and the architecture of the tissue containing the tumor originating cells. This does not mean that the omitted processes are negligible. Instead, the relatively small effective replacement rate obtained implies that the processes succeeding the clonal conversion of crypts set the time scale of the incidence rates. However, the results suggest that the shape and composition of the incidence rates may be determined by the structure of the tissue hosting the tumor-originating cells and the competition dynamics inside the crypts.

Note that in contrast to most previous pretumor models, which usually introduce four or more parameters that are calibrated using the epidemiological age-specific cancer incidences, both for colon [2, 17, 24, 29] or other types of cancer [16, 18, 20, 25, 27, 28, 30], our model is solely based on directly measurable parameters, which are already known from clinical and biological studies on the colon independent of the epidemiological incidence rates. Consequently, the model only requires a single fit parameter, the effective replacement rate, which both sets the time scale of the niche-competition as well as compensates for the neglected time-span between fixation in the niche and clinical detection.

The model is additionally applied to rectal and gastric cancer, whose tumor-originating cells are also proposed to be compartmentalized into niches, but exhibit incidence rates considerably smaller than for colon cancer. For this we only adapt the number of niches while assuming the same ranges as for the colon for all other parameters. Despite this simplification, we observe reasonable correspondence between predicted and epidemiological incidences rates. Furthermore, the obtained effective replacement rates are similar for all three cases. In combination, this points towards similar dynamics of tumor development for colon, rectum and stomach.

Note that for colorectal cancer, two-step models have been considered before to capture the dynamics of adenomatous and malignant tumor cells [2, 22–24]. Furthermore, the distribution of adenoma and adenomacarcinoma sizes at different ages has been inferred from a model [24], clonal expansion dynamics of colorectal cancer has been derived from intra-tumor heterogeneity [59] and even the competition in colonic crypts has been discussed [22, 23]. In contrast to these models, we explicitly incorporate competition of benign tumor cells with wild-type cells taking into account their spatial structure, while neglecting other processes considered in these models. The agreement of the prediction of our model and the epidemiological incidence rates emphasizes the dominant role that competition in niches may play in tumor development. This is interesting in the context of previous investigations of colorectal and gastric cancer using a multistage clonal expansion (MSCE) model, which suggest that the form of the age-specific incidence rates is determined by the dynamics until the fixation of tumor cells, while the growth dynamics after fixation only cause a slight time shift [18]. Future approaches could incorporate the model of crypt competition as process of tumor initiation into the MSCE model and examine how this affects the parameters of the model and their robustness.

Our model does not discern between patient subgroups such as sex, race, and cancer site. In principle, the model could be fitted to each of these subgroups, but note that there are considerable variations in the level of reporting across them, e.g., screening differences account for almost half of the disparity between colorectal incidences in white and black populations [37]. Moreover, the model assumes that all parameters are shared across the population, because the goal is not to predict individual cancer risks. For this, one would have to take into account individual predispositions regarding for instance genetics, immune system or lifestyle.

While the model is inspired by colonic crypts, it is potentially relevant for tumor development in a wide range of tissues. Firstly, the model can be applied to cancer types with similar spatial structure of the tumor-originating cells, as in the case of rectal or gastric cancer [63]. Secondly, the model may also apply to types of cancer whose tumor-originating cells are not explicitly compartmentalized into niches. The Moran model within the niche has already been used to estimate an upper limit for the number *N* of cells competing with each other from the fraction of clinically observed benign tumors for several types of cancer [34]. This so called competition range *N* is surprisingly small for a wide range of tumor types, less than 3000 cells, even for tumors in tissue without explicit stem cell compartments like the crypts. Note that for a large competition range *N* the competition between the malignant tumor cells and the other cells in the niche may not be negligible anymore, but increase the time scale until clonal conversion in the niche. Thus, the model has to be extented in the future by this competition to be applicable to types of cancer with large competition ranges, such as hepatocellular carcinoma [65] or glioblastoma [66].

Application of the model of niche competition to cancer in tissues other than the colon is reasonable for two more reasons: Firstly, most other solid tumors have an age-specific incidence qualitatively very similar to colon carcinoma [29]. Secondly, incidence rates resulting from the model share typical features with incidence rates from multistage clonal expansion models [16, 17, 21], which already achieve good matches with epidemiological data for several types of cancer [15–21]. Note that for most tissues, much less is known about tumor initiation and development until detection than for the colon. Applying the model to such types of cancer may give additional insight into the hardly observable dynamics of early cancer development.

## Supporting information

S1 Text Supporting figures. The Supporting Information text provides details on the Moran model, the numerical methods, and a systematic study on the effect of colorectal screening on the correspondence between model prediction and epidemiological incidence rates.
(PDF)
