# Supplementary material for: Modeling age-specific incidence of colon cancer via niche competition
Source: PLoS Comput Biol. 2022 Aug 19;18(8):e1010403. doi: 10.1371/journal.pcbi.1010403 (PMC9432715; doi:10.1371/journal.pcbi.1010403)
Supplement: S1 Text — The Supporting Information text provides details on the Moran model, the numerical methods, and a systematic study on the effect of colorectal screening on the correspondence between model prediction and epidemiological incidence rates. (PDF) [file pcbi.1010403.s001.pdf]

Supporting Information:
Modeling age-specific incidence of colon cancer via niche competition

Steffen Lange[1,2*], Richard Mogwitz[2], Denis Hünniger[1,2], Anja Voß-Böhme[1,2]

1 DataMedAssist, HTW Dresden, 01069 Dresden, Germany
2 Faculty of Informatics/Mathematics, HTW Dresden - University of Applied Sciences, 01069 Dresden

* steffen.lange@tu-dresden.de

A Moran Model of niche competition

We use a three-type Moran model with mutation to quantify the stem cell dynamics in the niche of a single colonic crypt as described in the main manuscript. The niche consists of N cells, each of which may be wild-type, benign or malignant. A cell in the niche is replaced by the offspring of another cell with replacement rate $N \cdot \lambda$, where λ is the replacement rate per stem cell. At replacement the offspring can additionally acquire mutations, i.e. a wild-type cells mutates with probability $u, 0 \leq u < 1$ into a benign tumor cell and a benign tumor cell mutates with probability $v, 0 \leq v < 1$ into malignant tumor cell. Formally, the model is a Markov process $(X_t)_{t\geq 0}$ on the state space $A = \{0, 1, 2, \ldots, N, E\}$, see sketch in Fig A in S1 Text for an illustration. Here, the states $0, \ldots, N$ correspond to the number of benign tumor cells in the niche, while the remaining cells in the niche are wild-type stem cells, and the state E represents the presence of a single malignant tumor cell in the niche. The dynamics is determined by a rate matrix $Q := (q(k,l))_{k,l\in A} \in R^{A\times A}$. For the space-free limit, i.e., when every cell is able to replace any other cell in the niche, the rate matrix Q_{sf} is given by

$$Q_{\text{sf}} = \begin{cases} q(k,k-1) & = \lambda \dfrac{k(N-k)}{N-1}(1-u) & ,1 \leq k \leq N-1 \\ q(k,k+1) & = \lambda \left(\dfrac{(N-k)(N-k-1)}{N-1} u + \dfrac{k(N-k)}{N-1}(1-v) \right) & ,0 \leq k \leq N-1 \\ q(k,k) & = -\displaystyle\sum_{i=0}^{N+1} q(k,i) & ,0 \leq k \leq E \\ q(k,E) & = \lambda k v & ,1 \leq k \leq N-1 \\ q(k,l) & = 0 & ,\text{else} \end{cases} . \tag{S1}$$

The factors containing N and k result from combinatorics [1–3]. The other geometric limiting case is the $1D$ model in which cells are considered to be ordered on a ring, i.e. each cell can only replace one of its two neighbors. Analogously, the rate matrix Q_{1D} for this case is given by [1, 3]

$$Q_{\text{1D}} = \begin{cases} q(0,1) & = \lambda N u & , \\ q(k,E) & = \lambda k v & ,1 \leq k \leq N-1 \\ q(k,k+1) & = \lambda(1-v) + O(u) & ,1 \leq k \leq N-1 \\ q(k,k-1) & = \lambda(1-u) & ,1 \leq k \leq N-1 \\ q(k,k) & = -\displaystyle\sum_{i=0}^{N+1} q(k,i) & ,0 \leq k \leq E \\ q(k,l) & = 0 & ,\text{else} \end{cases} , \tag{S2}$$

where, due to $u \ll 1$, we neglect the term $O(u)$, which describes an additional benign mutation of a wild type cell. This reasonable approximation implies that benign mutants only occur on a single connected interval of the ring of cells. A real crypt will have some geometric restrictions that lie in between these to limiting cases Q_{1D} and Q_{sf}.

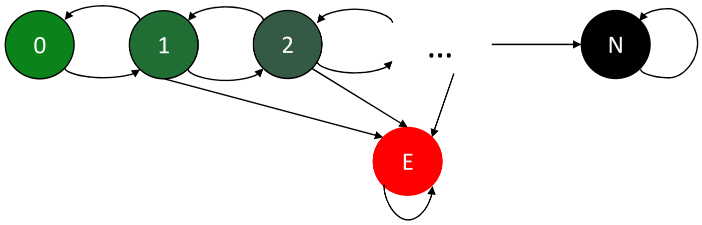

Fig A. Sketch of the markov chain modeling the competition within a niche. The transition rates corresponding to the arrows are expressed in Eq. (S1) and Eq. (S2)

For either rate matrix Q, the dynamics of the niche, i.e. the distribution $\mathbf{p} = (p_0, p_1, \dots, p_{N-1}, p_N, p_E) \in [0,1]^A$ on the state space A at age t which evolved from an initial state $\mathbf{p}(0)$ at age 0, is given by

$$\frac{\mathrm{d}}{\mathrm{d}t}\mathbf{p}(t) = \mathbf{p}(t)Q \tag{S3}$$

which yields the solution

$$\mathbf{p}(t) = \mathbf{p}(0)\exp(Qt), \tag{S4}$$

for $t \geq 0$, with the transition matrix $\exp(Qt)$ given by a matrixexponential function. The initial state is set to $\mathbf{p}(0) := (1, 0, \dots, 0)$, i.e. the niche starts at birth with only wild-type cells in the crypt. The probability $P_a(t)$ that the complete niche consists of benign tumor cells until age t is

$$P_a(t) = (\mathbf{p}(0)\exp(Qt))_N \tag{S5}$$

and the probability $P_c(t)$ that the first malignant cells emerges until age t is

$$P_c(t) = (\mathbf{p}(0)\exp(Qt))_E \ . \tag{S6}$$

We further list some analytic and empiric properties of the model, which demonstrate that the incidence rates generated by the model display features typical for multistage clonal expansion models, whose incidence rates transition (from young to old age) from power-law to exponential to linear to constant asymptote [4–6]: Previously, an analytic expression for the asymptotic absorbtion probabilities $\lim_{t\to\infty} P_a(t)$ and $\lim_{t\to\infty} P_c(t)$ into state N and state E, respectively, has been derived [2,3]. These asymptotic probabilities are predominantly determined by the relation of the niche size N and the probability v to progress from a benign tumor cell to a malignant one. A small niche $N\sqrt{v} \ll 1$ ($N\sqrt[3]{v} \ll 1$ for $1D$ model) is primarily absorbed in the benign state, whereas a large niche $N\sqrt{v} \gg 1$ ($N\sqrt[3]{v} \gg 1$ for $1D$ model) is primarily absorbed in the malignant state E, while intermediate niche sizes lead to finite probabilities for both states. This property has already been exploited to estimate upper limits for the effective niche sizes of several types of cancer from their recored benign tumor fraction [3]. Furthermore, the probability $\tilde{S}(t) = (1 - P_c(t) - P_a(t))$ to not be in either state N or E until time t decreases exponentially $\tilde{S}(t \gg 1) \sim \exp(\alpha t)$ for sufficiently large times t, where the rate $\alpha < 0$ is the largest eigenvalue of the rate matrix that results from limiting the rate matrix Q only to the non-absorbing states $\{0, 1, 2, \dots, N-1\}$. Since the probability $\tilde{S}(t)$ is qualitatively very similar to the survival probability $S(t)$ in Eq. (1), one may infer that the age-specific incidence rates $R(t)$ generated by the model according to Eq. (2) should become constant asymptotically $R(t \gg 1) \approx |\alpha|K$. Moreover, the probability of tunneling progression $P_c(t)$, whose contribution is maximal at young ages, follows a power law $P_c(t) \sim t$. This is in accordance with the argument of Armitage and Doll, that in a pure multistage model the incidence rates follow a power

law $R(t) \sim t^{\beta-1}$, with the number β of mutations necessary for a cell to become malignant [7,8], which is $\beta = 2$ in our model. In contrast, we observe numerically that the probability of sequential progression $P_a(t)$ matches the cumulative distribution function of a Weibull distribution $P_a(t) = 1 - \exp\left(-at^b\right)$, which implies for barely changing survival probabilities $S(t) \approx 1$, see Eq. (2),

$$R(t) = -\frac{\mathrm{d}}{\mathrm{d}t} \ln S(t) = -\frac{\frac{\mathrm{d}}{\mathrm{d}t} S(t)}{S(t)} \underset{S(t)\approx 1}{\approx} -\frac{\mathrm{d}}{\mathrm{d}t} S(t) \tag{S7}$$

that the contribution of the sequential progression to the incidence rates follows a Weibull distribution. Note that it is common for models of cancer development that the predicted incidence rates (or hazard function) effectively represent a Weibull distribution [9–14]. The assumption $S(t) \approx 1$ is reasonable, e.g., for colon cancer, since colon cancer is overall a relatively minor cause of death and has a low lifetime incidence.

B Fit of replacement rate

We determine the least known parameter, the effective replacement rate λ, by fitting the model to the epidemiological age-specific incidence rates $R_{\mathrm{SEER}}(t)$. First, we compute for given set of parameters N, u, v, K, and γ from the literature the survival probability $S(T)$ of the model in dimensionless time $T = t/\lambda$, using Eq. (1), Eq. (S5), and Eq. (S6) up until a sufficient long time $T_{\max}$ such that the probability $1 - S(T_{\max})$ is larger than the lifetime risk of colon cancer ($\geq 4.8\%$ [15]) to ensure that the corresponding incidence rates $R(T)$, see Eq. (2), cover the range of ages of the epidemiological data.

When the predicted $R(t)$ and epidemiological rates $R_{\mathrm{SEER}}(t)$ can be matched very close at all ages, the rate λ can be directly obtained by matching the corresponding survival probabilities $S(t)$, . Since the probabilities $S(t)$ are monotonously decreasing with age t, we can identify each data point $(t_i, S_{\mathrm{SEER}}(t_i))$ to a simulated point $(T, S(T))$ with $S_{\mathrm{SEER}}(t_i) = S(T)$. Thus, each data point i gives an estimate of the replacement rate $\lambda_i = T/t_i$ and we fit the replacement rate as their mean $\lambda =< \lambda_i >$. The standard deviation $\sqrt{< (\lambda_i - < \lambda_i >)^2 >}$ of the replacement rates may then serve as a measure of the quality of a single fit.

However, since deviations between predicted and epidemiological rates predicted $R(t)$ and epidemiological rates $R_{\mathrm{SEER}}(t)$ may cancel out in the corresponding survival probabilities, it is better to fit the rates directly. We use non-linear least-square minimization provided by the python package *lmfit* [16] to obtain the effective rate λ by minimizing the residuum

$$R_{\mathrm{SEER}}(t_i) - R(t_i) = R_{\mathrm{SEER}}(t_i) + \frac{\mathrm{d}}{\mathrm{d}t} \ln S(T = \lambda \cdot t_i) \qquad \forall t_i \ . \tag{S8}$$

For a given parameter set N, K, u, v, γ the goodness-of-fit χ^2 is quantified by the relative square deviation between the rescaled rate $R(t)$ of the model and the data $R_{\mathrm{SEER}}(t_i)$

$$\chi^2 = \sum_i \frac{\left[R_{\mathrm{SEER}}(t_i) - R(t_i)\right]^2}{R^2_{\mathrm{SEER}}(t_i)} \ . \tag{S9}$$

For the variation of the parameters N, K, u, v, γ the effective replacement rate λ is reported as average and standard deviation of the ensemble of fits.

C Adjustments for colorectal screening

The SEER data for colorectal cancer is fraught with two important secular trends, i.e., a downward trend of incidences at ages above 55 due to widespread colorectal screening and resection [18–22] and an upward trend of incidences of early onset colorectal cancer for ages below 50, whose origin is less understood but has been attributed to both life-style changes [18,23] as well as the detection of prevalent subclinical cases [23,24]. The incidences exhibit these age-group-dependent trends both over calendar and birth year, see Fig B in S1 Text, especially a strong birth cohort effect for younger ages. While these

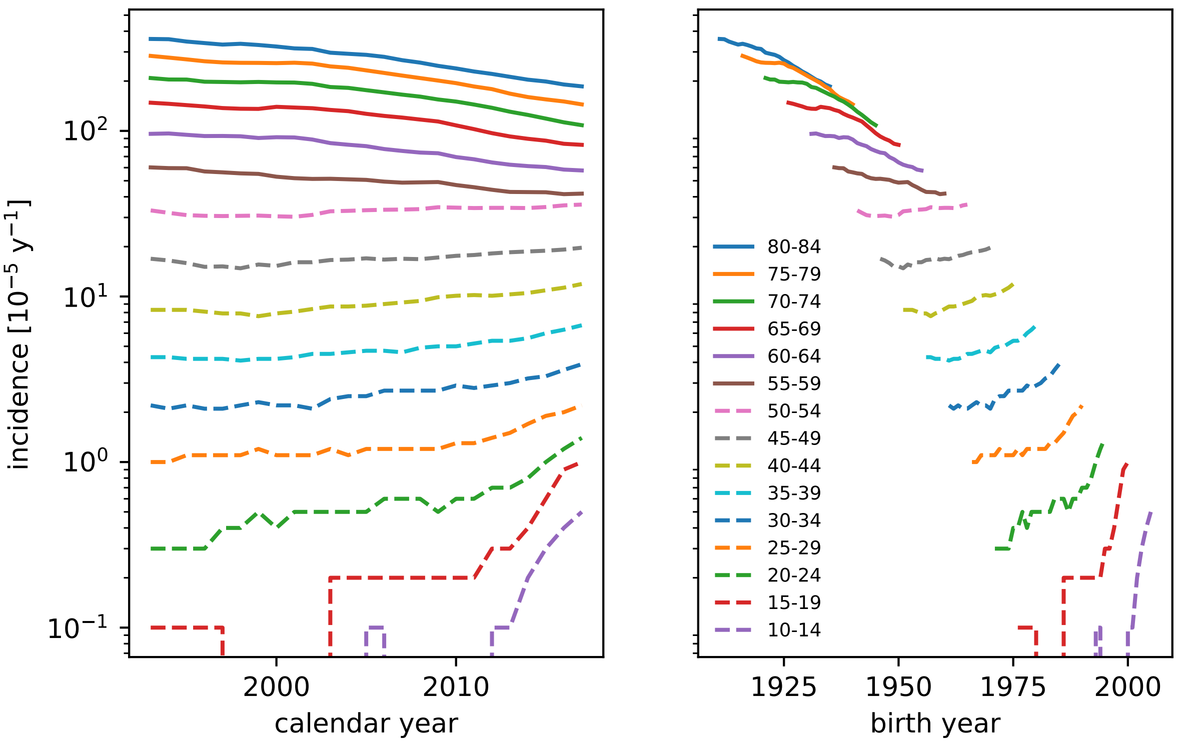

Fig B. Secular trends for colon cancer. Incidence rates of colon cancer shown as function of calendar year (left panel) and birth year (right panel) based on the archive of the SEER database 1993-2017 [17]. While pronounced secular trends are visible, they do not affect all age groups equally. Rather rates are increasing for age groups below 55 (dashed lines) years, while increasing above (solid lines).

trends may be indirectly compensated for by age-period-cohort (APC) epidemiological models [4, 25–28], such statistical adjustments do not allow to determine what the incidence rates would be without these external effects. Consequently, such adjustments are rarely invoked when comparing incidence rates to biological models of cancer development, in particular for colon cancer [9, 12, 29–31]. One approach could be to perform the APC adjustments, properly anchored for identifiability, on the relevant rates of the biological model, e.g., the replacement rate λ, to identify which parameters are most sensitive to the secular trends. Our approach is to directly incorporate independent estimates of the impact of colorectal screening.

While the exact quantification of the mentioned external effects is not possible, we consider several reasonable adjustments of the incidence rates from the SEER 2017 dataset to account for the effect of colorectal cancer screening. First of all we present the original incidences for colon and rectum from the SEER 2017 database in Fig C in S1 Text and Fig F in S1 Text along with the incidences from the previous years $1993-2017$ for illustration of the age-dependent secular trends. The first adjustment is based on estimates that the annual incidence of colorectal cancer at ages > 50 between 2000 and 2015 is reduced on average by $\sim 25\%$ due to screening [21]. Thus, the colon and rectal incidences displayed Figs. 1 and 4 correspond to the SEER database of 2017, for which the rates of all age groups above 55 years have been increased by a factor $4/3$. The second adjustment is based on the estimate that the impact of screening colonoscopy is modest in the age group $55-64$ and increases for older age groups [32].

From the predictions of Ref. [32] we obtain roughly an 8% reduction of annual incidences for the age group $55-64$, 28% for age group $65-74$, and 37% for the age group $75-84$. We assign these percentages to the ages 60, 70, and 80, respectively, and linearly interpolate the annual reductions between these ages, which we use to again adjust the results of the SEER database of 2017 in Fig D in S1 Text and Fig G in S1 Text. Finally, as a limit case we set the incidences above 55 year to the ones reported in the SEER 1993 database, see Fig E in S1 Text and Fig H in S1 Text.

For the colon, we find for each adjustment (and without adjustment) a similar correspondence between the epidemiological incidence rates and our model prediction. Mostly, the fitted effective replacement rate for a particular parameter set is modulated by the choice of the adjustment. For the rectum, the model displays a better correspondence with the incidence rates strongly adjusted for screening effects, see Fig G in S1 Text and Fig H in S1 Text. Note that the risk reduction is known to vary by subsite of the colon and rectum [32] while our estimates of this risk reduction are based on colorectal cancer [20, 21, 32], whose incidences are dominated by incidences of colon cancer.

Note that at ages younger than 50 colorectal screening occurs rarely and instead the rates may be subject to two opposing effects: On the one hand, the apparent rise in incidences below 50 years may be attributed to life-style changes [18, 23] an effect not incorporated in our biological model. On the other hand, recent results support the presence of a large undetected preclinical case burden < 50 years, which is not reflected in the rates of colorectal cancer observed in the SEER registries [24]. The latter is consistent with the fact, that people younger than 55 are more likely to be diagnosed with late-stage disease, largely due to sometimes for years delayed follow-up of symptoms [23]. This implies that cancer incidences are either missed at younger ages or wrongfully assigned to older age groups, whose rates they barely impact as the age-specific rates increase significantly with age. Since the net impact of these two opposing effects is unkown, we do not adjust incidence rates below 55 years, similar to Ref. [27].

Note that the secular trends for gastric cancer are less pronounced compared to colorectal cancer, see Fig I in S1 Text, and thus are used unadjusted from the SEER 2017 database.

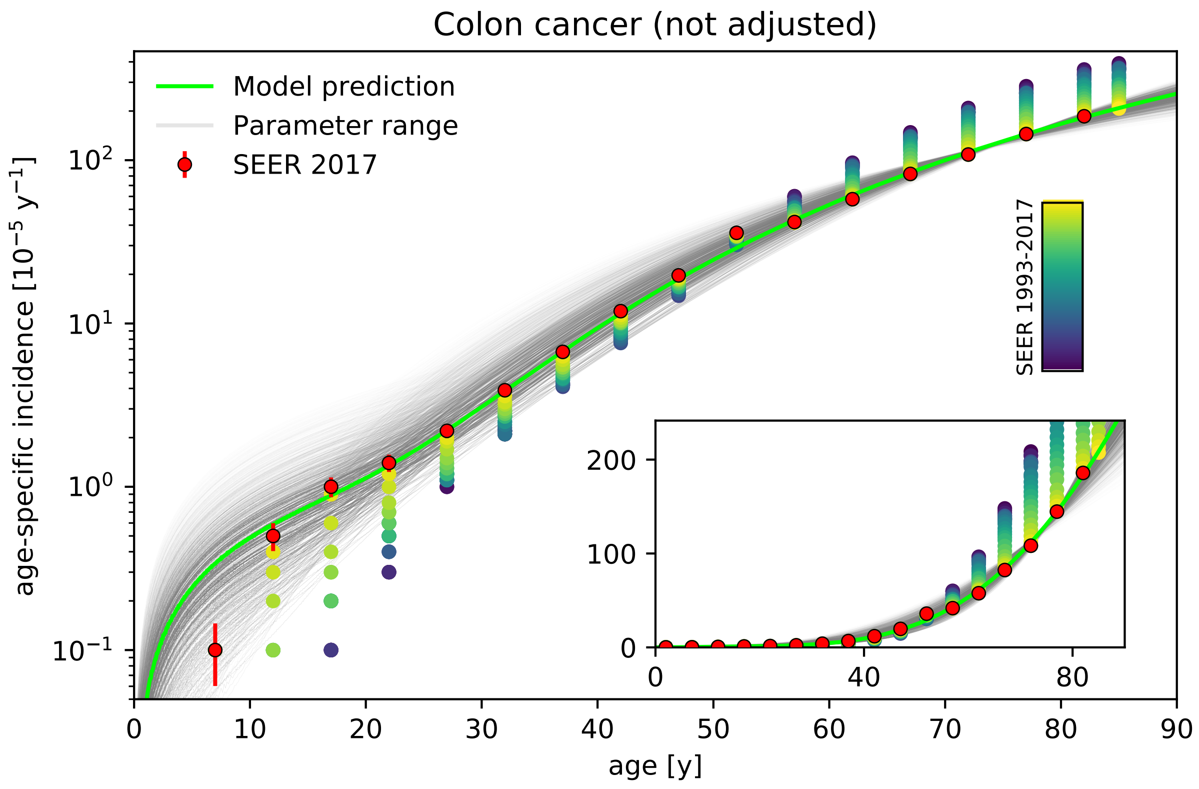

Fig C. Model comparison to colon data without adjustments for colorectal screening. Age-specific incidence rates of colon cancer predicted by the model and epidemiological data (SEER database [15] unadjusted, displayed curves and points analogous to Fig. 1). As illustration of secular trends, the epidemiological rates from the archive of the annual SEER database [17] are displayed by points colored according to the release year of the corresponding report from 1993 (dark blue) to 2017 (yellow). Exemplary parameter set with $N = 8$, $K = 2 \cdot 10^7$, $u = 1.75 \cdot 10^{-6}$, $v = 4.4 \cdot 10^{-6}$, and $\gamma = 9.4\%$ is highlighted in green. The effective replacement rate λ covers a range $0.01 - 0.06$ y^{-1} per stem cell with an average $\lambda = 0.02 \pm 0.01$ y^{-1} ($\lambda = 0.02$ y^{-1} per stem cell for the green curve).

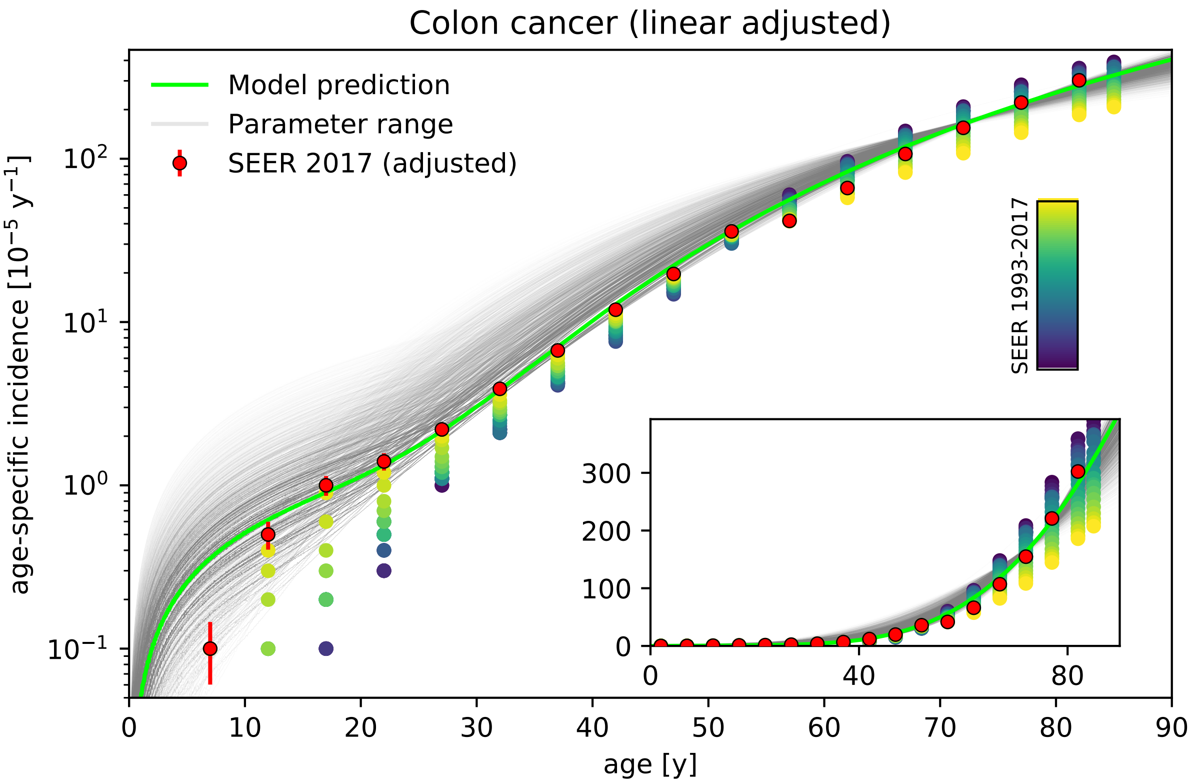

Fig D. Model comparison to colon data adjusted for colorectal screening by incrementally increased rates. Age-specific incidence rates of colon cancer predicted by the model and epidemiological data (SEER database [15] adjusted by incrementally increased rates ≥ 55 years, see text for details, displayed curves and points analogous to Fig. 1). As illustration of secular trends, the epidemiological rates from the archive of the annual SEER database [17] are displayed by points colored according to the release year of the corresponding report from 1993 (dark blue) to 2017 (yellow). Exemplary parameter set with $N = 9$, $K = 1.5 \cdot 10^7$, $u = 4.4 \cdot 10^{-6}$, $v = 1.75 \cdot 10^{-6}$, and $\gamma = 9.4\%$ is highlighted in green. The effective replacement rate λ covers a range $0.01 - 0.07$ y^{-1} per stem cell with an average $\lambda = 0.025 \pm 0.01$ y^{-1} ($\lambda = 0.022$ y^{-1} per stem cell for the green curve).

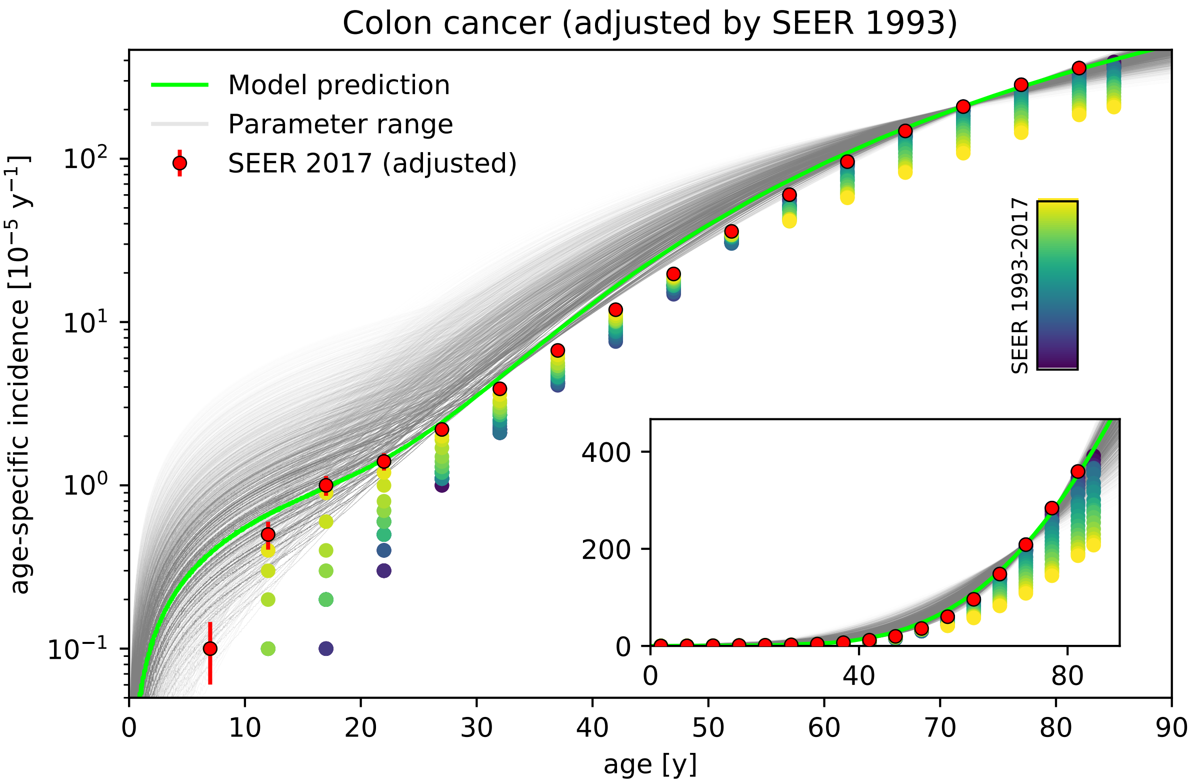

Fig E. Model comparison to colon data adjusted for colorectal screening by SEER 1993 database. Age-specific incidence rates of colon cancer predicted by the model and epidemiological data (SEER database [15] adjusted by rates from SEER 1993 [17] for ≥ 55 years, displayed curves and points analogous to Fig. 1). As illustration of secular trends, the epidemiological rates from the archive of the annual SEER database [17] are displayed by points colored according to the release year of the corresponding report from 1993 (dark blue) to 2017 (yellow). Exemplary parameter set with $N = 10$, $K = 2 \cdot 10^7$, $u = 1.75 \cdot 10^{-6}$, $v = 1.75 \cdot 10^{-6}$, and $\gamma = 9.4\%$ is highlighted in green. The effective replacement rate λ covers a range $0.01 - 0.09$ y^{-1} per stem cell with an average $\lambda = 0.03 \pm 0.01$ y^{-1} ($\lambda = 0.03$ y^{-1} per stem cell for the green curve).

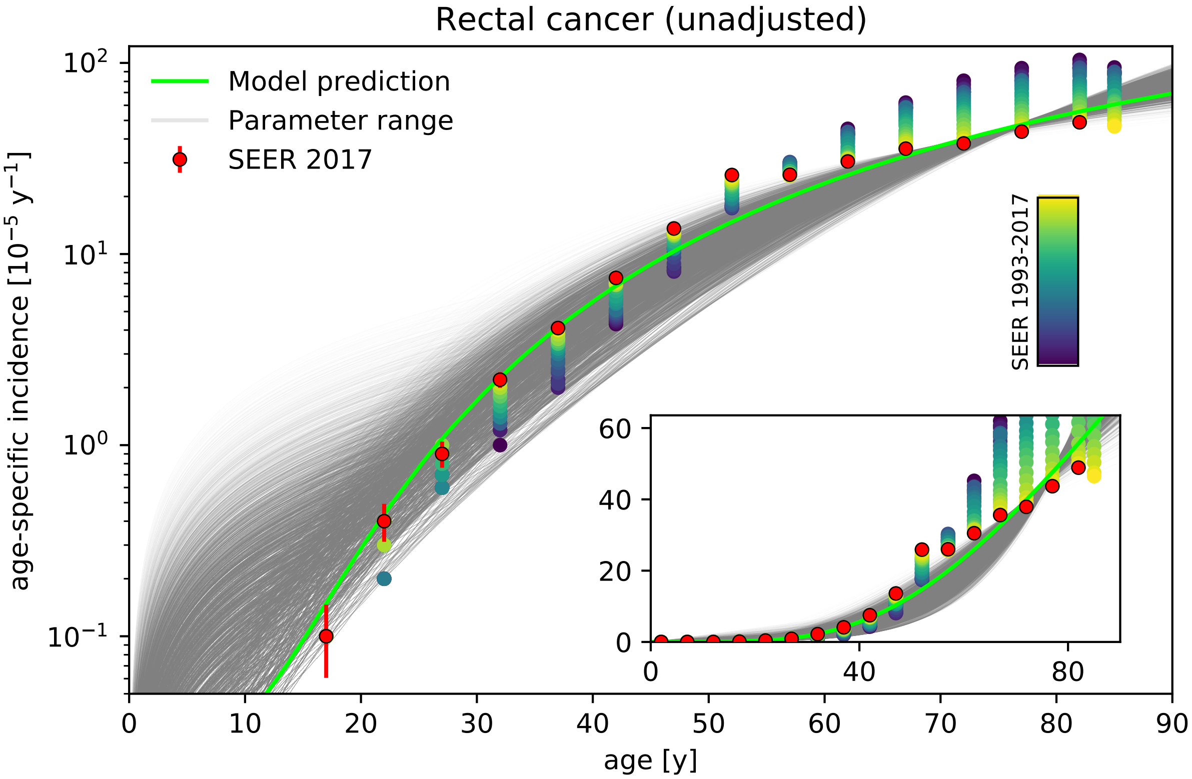

Fig F. Model comparison to rectum data without adjustments for colorectal screening. Age-specific incidence rates of rectal cancer predicted by the model and epidemiological data (SEER database [15] unadjusted, displayed curves and points analogous to Fig. 1). As illustration of secular trends, the epidemiological rates from the archive of the annual SEER database [17] are displayed by points colored according to the release year of the corresponding report from 1993 (dark blue) to 2017 (yellow). Exemplary parameter set with $N = 8$, $K = 10^6$, $u = 1.75 \cdot 10^{-6}$, $v = 1.75 \cdot 10^{-6}$, and $\gamma = 5\%$ is highlighted in green. The effective replacement rate λ covers a range $0.01 - 0.16$ y^{-1} per stem cell with an average $\lambda = 0.04 \pm 0.02$ y^{-1} ($\lambda = 0.036$ y^{-1} per stem cell for the green curve).

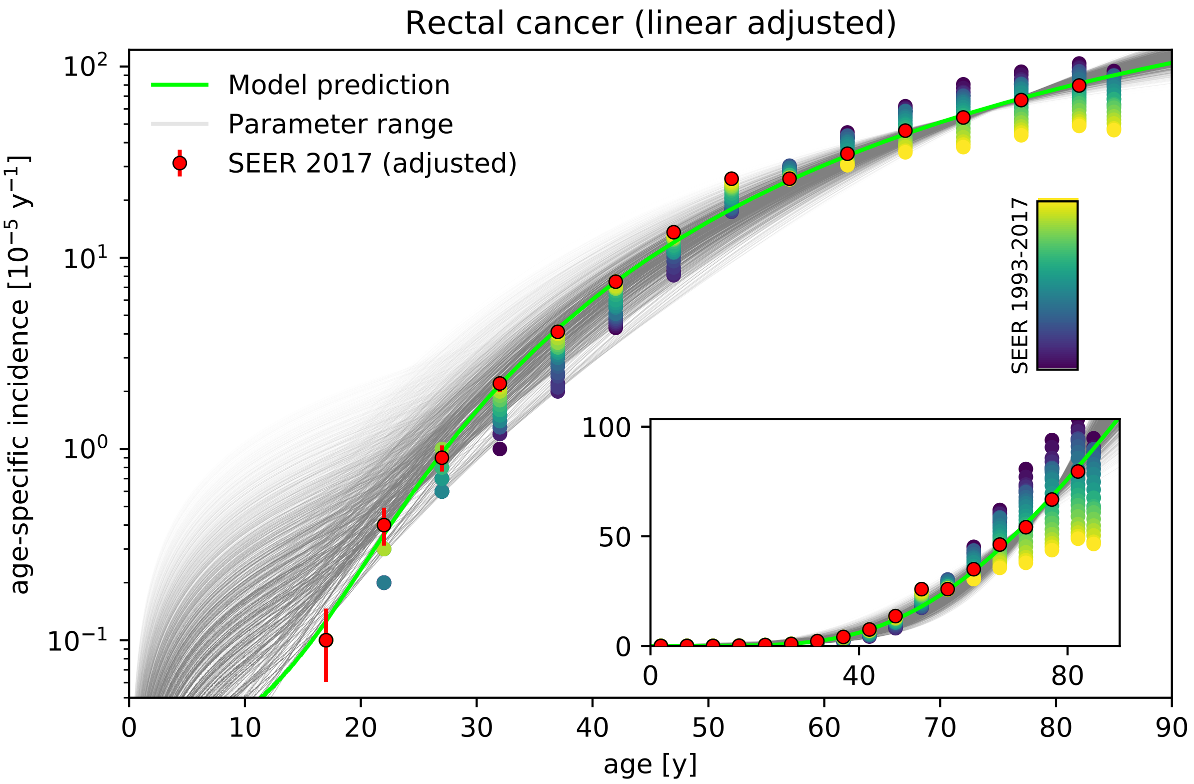

Fig G. Model comparison to rectum data adjusted for colorectal screening by incrementally increased rates. Age-specific incidence rates of rectal cancer predicted by the model and epidemiological data (SEER database [15] adjusted by incrementally increased rates ≥ 55 years, see text for details, displayed curves and points analogous to Fig. 1). As illustration of secular trends, the epidemiological rates from the archive of the annual SEER database [17] are displayed by points colored according to the release year of the corresponding report from 1993 (dark blue) to 2017 (yellow). Exemplary parameter set with $N = 9$, $K = 10^6$, $u = 1.75 \cdot 10^{-6}$, $v = 1.75 \cdot 10^{-6}$, and $\gamma = 9.4\%$ is highlighted in green. The effective replacement rate λ covers a range $0.01 - 0.12$ y^{-1} per stem cell with an average $\lambda = 0.04 \pm 0.02$ y^{-1} ($\lambda = 0.038$ y^{-1} per stem cell for the green curve).

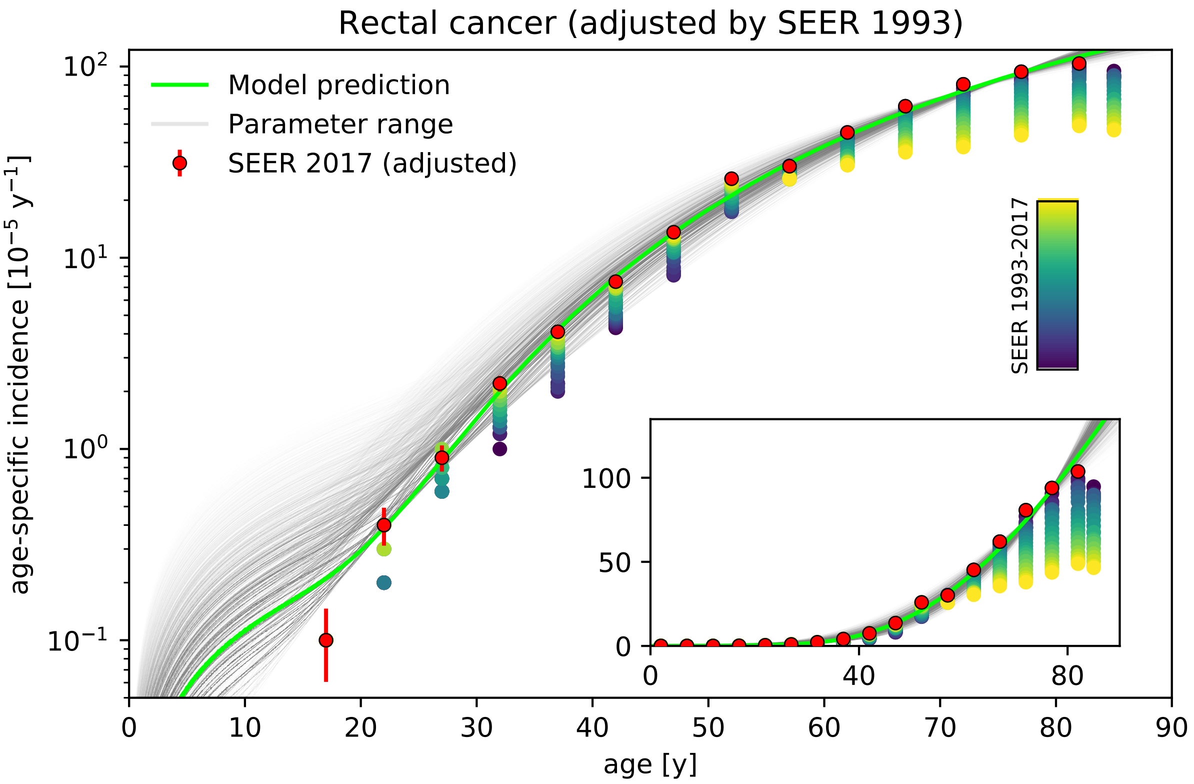

Fig H. Model comparison to rectum data adjusted for colorectal screening by SEER 1993 database. Age-specific incidence rates of rectal cancer predicted by the model and epidemiological data (SEER database [15] adjusted by rates from SEER 1993 [17] for ≥ 55 years, displayed curves and points analogous to Fig. 1). As illustration of secular trends, the epidemiological rates from the archive of the annual SEER database [17] are displayed by points colored according to the release year of the corresponding report from 1993 (dark blue) to 2017 (yellow). Exemplary parameter set with $N = 12$, $K = 10^6$, $u = 1.75 \cdot 10^{-6}$, $v = 1.75 \cdot 10^{-6}$, and $\gamma = 9.4\%$ is highlighted in green. The effective replacement rate λ covers a range $0.01 - 0.12$ y^{-1} per stem cell with an average $\lambda = 0.043 \pm 0.02$ y^{-1} ($\lambda = 0.055$ y^{-1} per stem cell for the green curve).

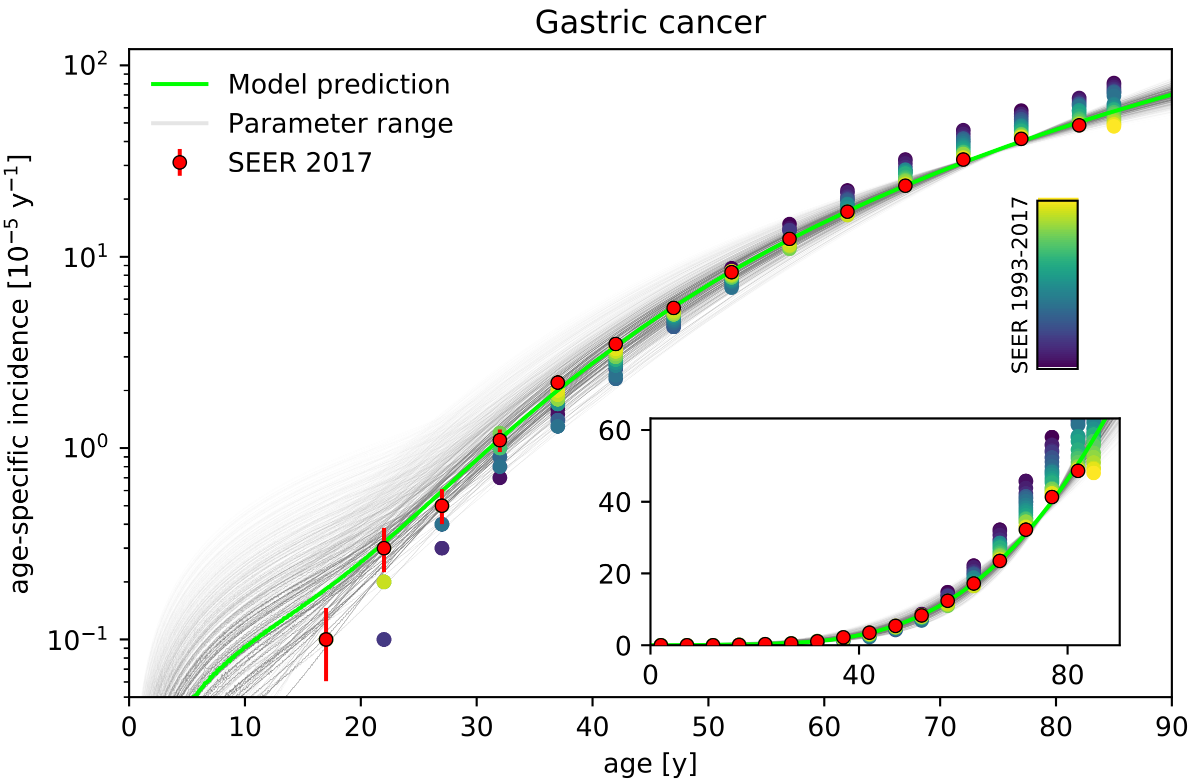

Fig I. Relation to trends over calendar year for gastric cancer. Age-specific incidence rates of gastric cancer predicted from the model and the epidemiological data as in Fig. 3. As illustration of secular trends, the epidemiological rates from the archive of the annual SEER database [17] are displayed by points colored according to the release year of the corresponding report from 1993 (dark blue) to 2017 (yellow). While qualitatively the same trends at younger and older age as for colon and rectal cancer are visible, these trends are significantly smaller for gastric cancer.

References

1. Komarova NL. Spatial Stochastic Models for Cancer Initiation and Progression. Bull Math Biol. 2006;68(7):1573–1599. doi:10.1007/s11538-005-9046-8.

2. Buder T, Deutsch A, Klink B, Voss-Böhme A. Model-Based Evaluation of Spontaneous Tumor Regression in Pilocytic Astrocytoma. PLoS Comput Biol. 2015;11(12):e1004662. doi:10.1371/journal.pcbi.1004662.

3. Buder T, Deutsch A, Klink B, Voss-Böhme A. Patterns of Tumor Progression Predict Small and Tissue-Specific Tumor-Originating Niches. Front Oncol. 2019;8. doi:10.3389/fonc.2018.00668.

4. Meza R, Jeon J, Renehan AG, Luebeck EG. Colorectal Cancer Incidence Trends in the United States and United Kingdom: Evidence of Right- to Left-Sided Biological Gradients with Implications for Screening. Cancer Res. 2010;70(13):5419–5429. doi:10.1158/0008-5472.CAN-09-4417.

5. Meza R, Jeon J, Moolgavkar SH, Luebeck EG. Age-specific incidence of cancer: Phases, transitions, and biological implications. Proc Natl Acad Sci USA. 2008;105(42):16284–16289. doi:10.1073/pnas.0801151105.

6. Brouwer AF, Meza R, Eisenberg MC. Parameter estimation for multistage clonal expansion models from cancer incidence data: A practical identifiability analysis. PLoS Comput Biol. 2017;13(3):e1005431. doi:10.1371/journal.pcbi.1005431.

7. Armitage P, Doll R. The Age Distribution of Cancer and a Multi-stage Theory of Carcinogenesis. Br J Cancer. 1954;8(1):1–12. doi:10.1038/bjc.1954.1.

8. Armitage P, Doll R. The age distribution of cancer and a multi-stage theory of carcinogenesis. Br J Cancer. 2004;91(12):1983–1989. doi:10.1038/sj.bjc.6602297.

9. Soto-Ortiz L, Brody JP. A theory of the cancer age-specific incidence data based on extreme value distributions. AIP Adv. 2012;2(1):011205. doi:10.1063/1.3699050.

10. Mdzinarishvili T, Sherman S. Weibull-like Model of Cancer Development in Aging. Cancer Inform. 2010;9:CIN.S5460. doi:10.4137/CIN.S5460.

11. Mdzinarishvili T, Gleason MX, Kinarsky L, Sherman S. A Generalized Beta Model for the Age Distribution of Cancers: Application to Pancreatic and Kidney Cancer. Cancer Inform. 2009;7:CIN.S3050. doi:10.4137/CIN.S3050.

12. Calabrese P, Tavaré S, Shibata D. Pretumor Progression: Clonal Evolution of Human Stem Cell Populations. Am J Pathol. 2004;164(4):1337–1346. doi:10.1016/S0002-9440(10)63220-8.

13. Grotmol T, Bray F, Holte H, Haugen M, Kunz L, Tretli S, et al. Frailty Modeling of the Bimodal Age-Incidence of Hodgkin Lymphoma in the Nordic Countries. Cancer Epidem Biomar. 2011;20(7):1350–1357. doi:10.1158/1055-9965.EPI-10-1014.

14. Brody JP. Age-Specific Incidence Data Indicate Four Mutations Are Required for Human Testicular Cancers. PLoS ONE. 2011;6(10):e25978. doi:10.1371/journal.pone.0025978.

15. Surveillance, Epidemiology, and End Results (SEER) Program (www.seer.cancer.gov) Research Data (1975-2017), National Cancer Institute, DCCPS, Surveillance Research Program, released April 2020, based on the November 2019 submission. `https://seer.cancer.gov/archive/csr/1975_2017/download_csr_datafile.php/sect_06_table.11.csv`; `https://seer.cancer.gov/archive/csr/1975_2017/download_csr_datafile.php/sect_24_table.07.csv`;.

16. Newville M, Stensitzki T, Allen DB, Ingargiola A. LMFIT: Non-Linear Least-Square Minimization and Curve-Fitting for Python; 2014. Available from: `https://zenodo.org/record/11813`.

17. Surveillance, Epidemiology, and End Results (SEER) Program (www.seer.cancer.gov) Cancer Statistics Review Archive (1993-2017), National Cancer Institute, DCCPS, Surveillance Research Program https://seer.cancer.gov/csr/previous.html;.

18. Siegel R, DeSantis C, Jemal A. Colorectal cancer statistics, 2014. CA: Cancer J Clin. 2014;64(2):104–117. doi:10.3322/caac.21220.

19. Bailey CE, Hu CY, You YN, Bednarski BK, Rodriguez-Bigas MA, Skibber JM, et al. Increasing Disparities in the Age-Related Incidences of Colon and Rectal Cancers in the United States, 1975-2010. JAMA Surg. 2015;150(1):17–22. doi:10.1001/jamasurg.2014.1756.

20. Kahi CJ, Imperiale TF, Juliar BE, Rex DK. Effect of Screening Colonoscopy on Colorectal Cancer Incidence and Mortality. Clin Gastroenterol Hepatol. 2009;7(7):770–775. doi:10.1016/j.cgh.2008.12.030.

21. Levin TR, Corley DA, Jensen CD, Schottinger JE, Quinn VP, Zauber AG, et al. Effects of Organized Colorectal Cancer Screening on Cancer Incidence and Mortality in a Large Community-Based Population. Gastroenterology. 2018;155(5):1383–1391.e5. doi:10.1053/j.gastro.2018.07.017.

22. Cardoso R, Zhu A, Guo F, Heisser T, Hoffmeister M, Brenner H. Incidence and Mortality of Proximal and Distal Colorectal Cancer in Germany. Dtsch Arztebl Int. 2021;118(16):281–287. doi:10.3238/arztebl.m2021.0111.

23. Siegel RL, Fedewa SA, Anderson WF, Miller KD, Ma J, Rosenberg PS, et al. Colorectal Cancer Incidence Patterns in the United States, 1974-2013. J Natl Cancer Inst. 2017;109(8):djw322. doi:10.1093/jnci/djw322.

24. Abualkhair WH, Zhou M, Ahnen D, Yu Q, Wu XC, Karlitz JJ. Trends in Incidence of Early-Onset Colorectal Cancer in the United States Among Those Approaching Screening Age. JAMA Netw Open. 2020;3(1):e1920407. doi:10.1001/jamanetworkopen.2019.20407.

25. Luebeck EG, Moolgavkar SH. Multistage carcinogenesis and the incidence of colorectal cancer. Proc Natl Acad Sci USA. 2002;99(23):15095–15100. doi:10.1073/pnas.222118199.

26. Moolgavkar SH, Meza R, Turim J. Pleural and peritoneal mesotheliomas in SEER: age effects and temporal trends, 1973-2005. Cancer Causes Control. 2009;20(6):935–944. doi:10.1007/s10552-009-9328-9.

27. Luebeck EG, Curtius K, Jeon J, Hazelton WD. Impact of Tumor Progression on Cancer Incidence Curves. Cancer Res. 2013;73(3):1086–1096. doi:10.1158/0008-5472.CAN-12-2198.

28. Brouwer AF, Eisenberg MC, Meza R. Age Effects and Temporal Trends in HPV-Related and HPV-Unrelated Oral Cancer in the United States: A Multistage Carcinogenesis Modeling Analysis. PLoS ONE. 2016;11(3):e0151098. doi:10.1371/journal.pone.0151098.

29. Kim KM, Calabrese P, Tavaré S, Shibata D. Enhanced Stem Cell Survival in Familial Adenomatous Polyposis. Am J Pathol. 2004;164(4):1369–1377. doi:10.1016/S0002-9440(10)63223-3.

30. Little MP, Vineis P, Li G. A stochastic carcinogenesis model incorporating multiple types of genomic instability fitted to colon cancer data. J Theor Biol. 2008;254(2):229–238. doi:10.1016/j.jtbi.2008.05.027.

31. Lang BM, Kuipers J, Misselwitz B, Beerenwinkel N. Predicting colorectal cancer risk from adenoma detection via a two-type branching process model. PLoS Comput Biol. 2020;16(2):e1007552. doi:10.1371/journal.pcbi.1007552.

32. Brenner H, Altenhofen L, Stock C, Hoffmeister M. Expected long-term impact of the German screening colonoscopy programme on colorectal cancer prevention: Analyses based on 4,407,971 screening colonoscopies. Eur J Cancer. 2015;51(10):1346–1353. doi:10.1016/j.ejca.2015.03.020.
